# Housing Correlates in Pregnant and Parenting Women Using Methamphetamine and Accessing Psychiatric Care

**DOI:** 10.3389/fpsyt.2022.890635

**Published:** 2022-05-27

**Authors:** Johannes Petzold, Laura Rehmet, Benjamin Weber, Maik Spreer, Maria Krüger, Ulrich S. Zimmermann, Maximilian Pilhatsch

**Affiliations:** ^1^Department of Psychiatry and Psychotherapy, Carl Gustav Carus University Hospital, Technische Universität Dresden, Dresden, Germany; ^2^Department of Addiction Medicine and Psychotherapy, Kbo-Isar-Amper-Klinikum München-Ost, Haar, Germany; ^3^Department of Psychiatry and Psychotherapy, Elblandklinikum Radebeul, Radebeul, Germany

**Keywords:** perinatal substance use, maternal drug use, pregnancy, socioeconomic deprivation, unstable housing, multimodal therapy, methamphetamine use disorder, drug addiction

## Abstract

**Background:**

Integrated care is a promising model for pregnant and parenting women with problems related to methamphetamine use. Yet more research is imperative to guide services for this vulnerable population as methamphetamine use contributes to housing instability, which is associated with heavier use and overdose death.

**Method:**

This prospective observational study analyzed how housing at discharge from psychiatric care was related to patient characteristics, program participation, and aftercare in 102 pregnant and/or parenting women.

**Results:**

Twelve of 23 women who were unstably housed at admission (three of six homeless) achieved stable housing by discharge from integrated care. Women were more likely unstably housed at discharge when unstably housed at admission, single, living apart from at least one minor, or when the other parent had a substance use disorder (*p* < 0.05). Unstably housed women at discharge were also more likely to have used social and inpatient services, and to transition to inpatient rehabilitation (*p* < 0.05). Among baseline characteristics, logistic regression identified unstable housing at admission (*OR* = 6.07) and being single (*OR* = 4.01) as the strongest unique contributors to unstable housing at discharge (*p* < 0.05).

**Conclusion:**

Unstably housed women and single women seem particularly at risk of remaining in precarious living conditions despite accessing integrated care for problems associated with methamphetamine use. Future work should investigate whether stronger partnerships with government and community agencies could be a way forward to help these women attain and maintain stable housing.

## Introduction

Global methamphetamine markets continue expanding while methamphetamine use has reached epidemic proportions in many parts of the world (1–3). This escalation threatens public health due to the related morbidity, mortality, and criminality (1–3). Methamphetamine use and unintended pregnancies can have detrimental consequences for women and their offspring, such as eclampsia and death (4–8). Newborns with prenatal methamphetamine exposure are at risk for withdrawal symptoms and developmental deficits, which include reduced weight, size, and head circumference (5–8). These children also exhibit more problems in later life, such as signs of attention-deficit hyperactivity disorder at school age (5–7). Pregnant and parenting women who use methamphetamine often struggle with polysubstance use, mental illness, socioeconomic deprivation, and single parenting (8–11). Compared with parents using other substances, parents using methamphetamine are more likely female, unemployed, not in a committed relationship, and without custody of their children (12). Moreover, parenting stress is even higher in methamphetamine-using mothers than fathers (13, 14). Despite the need for prevention and intervention, methamphetamine-using women experience limited pregnancy, pediatric, and mental healthcare (6, 8, 10, 11, 15). Barriers concern the awareness of services and care needs, availability, affordability, logistics, and legal implications regarding substance use and childcare (16, 17). Access is additionally hampered by stigmatizing attitudes toward methamphetamine use, substance use in pregnancy, socioeconomic adversity, and legal system involvement (16, 17). This accumulation of biopsychosocial stressors can fuel a vicious cycle of poor maternal and child health, substance use, adversity, and marginalization (6, 9, 18).

The concept “Mama denk an mich” (Mamadam, “Mommy think of me”) was developed to break this cycle by providing low-barrier care across disciplines and settings. Case management and coordination meetings integrate the Psychiatric, Obstetric, and Pediatric Departments at the Dresden University Hospital with child welfare and community substance use services. Care is mainly provided on an outpatient basis to promote psychosocial functioning and well-being in daily life.

Psychiatric care draws on the available evidence and best practice for the management of methamphetamine-related disorders (19). Patients consult with a psychiatrist and/or psychotherapist from several times a week to once a month as needed. Mamadam provides women-only group psychotherapy based on a manual that combines methamphetamine-specific psychoeducation, motivational interviewing, and cognitive behavior therapy (20–23). Hospital social workers partner with community and government agencies to aid patients in finances, work, and housing. Childcare is supported by establishing a crisis plan in case of relapse and by in-home assistance if needed. Supervised random substance screening is employed to promote treatment engagement. Inpatient care is also available, which provides additional group therapies, such as exercise classes, progressive muscle relaxation, occupational therapy, social skills training, and specific therapies for psychiatric comorbidities.

Previous studies described the Mamadam concept in detail and added to the evidence that pregnancy and parenthood provide opportunities to motivate change (13, 19, 24), thereby decreasing substance use and child removal (15, 25–27). Patients could be engaged in outpatient psychiatric treatment over months, but more research is imperative to evaluate and optimize care strategies. Studying housing instability is insightful as it may not only be a consequence (28, 29) but also a cause of substance use and even overdose death (30, 31). Moreover, meeting the basic need for stable housing is essential for effective social and professional reintegration. We thus investigated how housing at discharge from Mamadam was related to health and life circumstances at admission as well as healthcare utilization before, during, and after Mamadam. The goal was to gather information on what interventions may be beneficial and how best to integrate them to prevent and mitigate harms related to methamphetamine and other substance use during pregnancy and parenthood. These findings should inform care initiatives that are urgently needed to counter the impact of the global methamphetamine crisis on women and children.

## Methods

This prospective observational study aimed to identify baseline and treatment correlates of housing in pregnant and parenting women with methamphetamine use disorder according to the Diagnostic and Statistical Manual of Mental Disorders, Fifth Edition. We studied all 102 women who accessed psychiatric care within Mamadam since its start in 2016 and left or completed treatment before July 30, 2020. This naturalistic sample included the 73 women whose treatment adherence was previously analyzed (27). Adherence was categorized as early discontinuation (before implementing a care plan), partial completion of the program (late discontinuation), or successful completion (requiring stable abstinence).

Statistics were performed in SPSS 27 (IBM, Armonk, NY, USA), using two-sided tests, a significance level of 0.05, and data on all patients unless stated otherwise. We categorized women depending on whether they had control over their living arrangements at discharge as stably (own apartment, condominium, house) or unstably housed (supported transitional accommodation with children, living in others' homes, homeless). They were compared on baseline characteristics, Mamadam participation, and aftercare. Pearson's chi-square test (or Fisher's exact test when cell sizes were too small) and Bonferroni-adjusted pairwise comparisons were applied for categorical variables. We used Mann-Whitney U tests as histograms, normal quantile-quantile plots, and tests of normality demonstrated that continuous data were not normally distributed.

We quantified associations between variables that differed significantly between groups with the phi coefficient. To identify the variables with the strongest unique contributions to housing at discharge, we built one model with baseline and another with treatment variables. All variables that had complete data and met the assumptions of logistic regression were considered for forward stepwise selection using the likelihood ratio. Significant variables from these models were entered in a third model to discriminate how much variability of housing stability at discharge was explained by each variable when controlling for the others.

## Results

### Study Sample and Care Utilization

The naturalistic sample consisted of pregnant (*n* = 38) and/or parenting (*n* = 95) women with problems related to methamphetamine use. Of the 102 women, 79 were stably housed at admission to psychiatric care and 82 at discharge. Living arrangements and changes therein are detailed in Table 1. Table 2 displays baseline characteristics, program participation, and aftercare, stratified by housing at discharge.

**Table 1:** Housing at admission and discharge.

			**Housing at discharge**				
			**Stable**	**Unstable**			
			**Own**	**Supported**	**Others'**	**Homeless**	**Total**
Housing at admission	Stable	Own	70	9	0	0	**79**
	Unstable	Supported	4	2	1	0	**7**
		Others'	5	0	5	0	**10**
		Homeless	3	0	1	2	**6**
		Total	**82**	**11**	**7**	**2**	**102**

*Depending on whether women had control over their living arrangements, housing was considered stable (own apartment, condominium, house) or unstable (supported transitional accommodation with children, living in others' homes, homeless)*.

**Table 2:** Patient characteristics by housing at discharge.

	**Housing at discharge**		**Group differences**
	**Stable (*n* = 82)**	**Unstable (*n* = 20)**	
**Baseline**			
Age^B^	28.65 ± 5.84	28.90 ± 6.15	*U* = 850.50, *z* = 0.26, *p* = 0.797
Pregnant^B^	39.0	30.0	*X^2^* (1) = 0.560, *p* = 0.454^C^
Childless^B^	7.3	5.0	*X^2^* (1) = 0.135, *p* = 1.000^F^
Children^B^	2.06 ± 1.35	2.20 ± 1.54	*U* = 846.50, *z* = 0.23, *p* = 0.817
Mother-child separation (*n* = 95 mothers)	57.9	84.2	*X^2^* (1) = 4.524, *p* = 0.033^C^^,^^*^
Single^B^	45.1	80.0	*X^2^* (1) = 7.836, *p* = 0.005^C^^,^^*^
Stable housing^B^	85.4	45.0	*X^2^* (1) = 15.001, *p* < 0.001^F^^,^^*^
Employed^B^	12.2	10.0	*X^2^* (1) = 0.075, *p* = 1.000^F^
Criminal record (*n* = 97)			*X^2^* (2) = 4.134, *p* = 0.144^F^
None	62.8	42.1	
Fine or community service	20.5	21.1	
Prison sentence	16.7	36.8	
Years of regular methamphetamine use (*n* = 96)	6.71 ± 5.90	7.44 ± 6.84	*U* = 729.00, *z* = 0.25, *p* = 0.799
Prior withdrawal program^B^	47.6	65.0	*X^2^* (1) = 1.957, *p* = 0.162^C^
Current psychiatric comorbidity^#^			
Substance use disorder^B^	57.3	35.0	*X^2^* (1) = 3.214, *p* = 0.073^C^
Depressive disorder^B^	13.4	15.0	*X^2^* (1) = 0.034, *p* = 1.000^F^
Personality disorder^B^	17.1	25.0	*X^2^* (1) = 0.667, *p* = 0.521^F^
Attention-deficit hyperactivity disorder^B^	14.6	5.0	*X^2^* (1) = 1.342, *p* = 0.303^F^
Any	73.2	80.0	*X^2^* (1) = 0.395, *p* = 0.530^C^
Any except substance use disorder	41.5	60.0	*X^2^* (1) = 2.231, *p* = 0.135^C^
Other parent with substance use disorder (*n* = 88)^#^			*X^2^* (2) = 7.207, *p* = 0.026^F^^,^^*^
No	36.6	5.9	+
Abstinent	19.7	17.6	–
Yes	43.7	76.5	+
**Program participation**			
Adherence			*X^2^* (2) = 3.491, *p* = 0.157^F^
Early discontinuation^T^	19.5	15.0	
Partial completion	32.9	15.0	
Successful completion^T^	47.6	70.0	
Days^T^	198.38 ± 200.91	178.80 ± 174.06	*U* = 783.50, *z* = −0.31, *p* = 0.758
Inpatient care^T^	15.9	45.0	*X^2^* (1) = 8.074, *p* = 0.008^F^^,^^*^
Methamphetamine-specific group therapy^T^	72.0	70.0	*X*^2^ (1) = 0.030, *p* = 0.862^C^
Individual psychotherapy^T^	14.6	15.0	*X*^2^ (1) = 0.002, *p* = 1.000^F^
Social services^T^	46.3	80.0	*X^2^* (1) = 7.311, *p* = 0.007^C^^,^^*^
**Aftercare** ^†^			
Inpatient rehabilitation^T^	7.3	30.0	*X*^2^ (1) = 7.969, *p* = 0.012^F^^,^^*^
Community substance use services^T^	43.9	25.0	*X*^2^ (1) = 2.390, *p* = 0.122^C^

*Statistics were performed on data from all women (N = 102) unless stated otherwise, listing group M ± SD or percentages of women within the relevant housing category*.

B,T *Used in Table 4 displayed regression models*.

# *Tobacco use disorder was not recorded*.

† *Not recorded for others, such as primary care physicians, private psychiatrists, or child welfare services*.

C *Pearson's chi-square test*;

F *Fisher's exact test; U, Mann-Whitney U test*.

* *Significant at p < 0.05*.

*+/–, significant/non-significant at p < 0.05 in Bonferroni-adjusted post-hoc analyses*.

### Baseline Correlates of Housing at Discharge

Women were more likely unstably housed at discharge when single, unstably housed at admission, living apart from at least one minor, or when the other parent had a substance use disorder (*p* < 0.05). The majority of unstably housed women at discharge had a criminal record (prison sentence in over one-third), whereas almost two-thirds of stably housed patients had no record (prison sentence in one-sixth), yet there was no overall significant difference in criminal system involvement between groups. About 10% of women across groups were employed and about three-fourths had a current psychiatric comorbidity. Groups were also similar in age, being pregnant, being childless, number of minors, years of methamphetamine use, and prior withdrawal program participation (*p* > 0.05).

### Treatment Correlates of Housing at Discharge

Unstably housed women at discharge were more likely to have used social and inpatient services, and to transfer to inpatient rehabilitation (*p* < 0.05). Groups were comparable in attendance at individual and methamphetamine-specific group psychotherapies as well as in program adherence and duration (*p* > 0.05).

### Associations Between Correlates of Housing at Discharge

Table 3 presents associations among all baseline and treatment variables that differed significantly between stably and unstably housed women at discharge. Being single was significantly related to the other parent having a substance use disorder and trend-level related to unstable housing at baseline. These three baseline characteristics and living apart from at least one minor were each related to one or more variables of higher treatment intensity (accessing social services, inpatient care, inpatient rehabilitation), which in turn were positively intercorrelated (*p* < 0.05).

**Table 3:** Associations between correlates of housing at discharge.

	**Single^**B**^**	**Mother-child separation^**B**^**	**Unstable housing at baseline^**B**^**	**Other parent with substance use disorder^***T***^**	**Inpatient care^***T***^**	**Social work^***T***^**
Mother-child separation^B^	*r*_ϕ_ = 0.030 *p* = 0.771^C^					
Unstable housing at baseline^B^	*r*_ϕ_ = 0.190 *p* = 0.055^C^	*r*_ϕ_ = 0.126 *p* = 0.219^C^				
Other parent with substance use disorder^B^	*r*_ϕ_ = 0.326 *p* = 0.009^C^^,^^*^	*r*_ϕ_ = 0.202 *p* = 0.187^C^	*r*_ϕ_ = 0.225 *p* = 0.106^F^			
Inpatient care^T^	*r*_ϕ_ = 0.170 *p* = 0.086^C^	*r*_ϕ_ = 0.249 *p* = 0.015^C^^,^^*^	*r*_ϕ_ = 0.230 *p* = 0.026^F^^,^^*^	*r*_ϕ_ = 0.202 *p* = 0.191^F^		
Social work^T^	*r*_ϕ_ = 0.234 *p* = 0.018^C^^,^^*^	*r*_ϕ_ = 0.324 *p* = 0.002^C^^,^^*^	*r*_ϕ_ = 0.133 *p* = 0.180^C^	*r*_ϕ_ = 0.346 *p* = 0.005^C^^,^^*^	*r*_ϕ_ = 0.399 *p* < 0.001^C^^,^^*^	
Inpatient rehabilitation^T^	*r*_ϕ_ = 0.168 *p* = 0.089^C^	*r*_ϕ_ = 0.225 *p* = 0.051^F^	*r*_ϕ_ = 0.313 *p* = 0.005^F^^,^^*^	*r*_ϕ_ = 0.206 *p* = 0.167^F^	*r*_ϕ_ = 0.548 *p* < 0.001^F^^,^^*^	*r*_ϕ_ = 0.222 *p* = 0.025^C^^,^^*^

*Associations among baseline (B) and treatment (T) variables that were significantly related to housing at discharge (see Table 2). Variables were coded as 0 = no and 1 = yes, except other parent with substance use disorder (0 = no, 1 = abstinent, 2 = yes). Pearson's chi-square (C) and Fisher's exact tests (F) used data from all women (N = 102) unless involving variables with reduced sample sizes as detailed in Table 2*.

* *Significant at p < 0.05*.

### Predictors of Housing at Discharge

Table 4 lists the unique (net) contributions of selected baseline and treatment variables to housing at discharge (controlled for the other variables in the respective model). Among baseline characteristics (first model), being single and unstably housed predicted unstable housing at discharge (*p* < 0.05). Among treatment variables (second model), entering inpatient care predicted unstable housing at discharge (*p* < 0.05). When these three variables were tested together (third model), being single and unstably housed at baseline retained significance, whereas inpatient care reached trend-level significance.

**Table 4:** Logistic regression models with correlates of housing at discharge.

	**B (SE)**	**OR**	***p***
**First model built by forward stepwise selection of baseline characteristics**^**B**^ **X**^**2**^ **(2)** **=** **18.599**, ***p*** **<** **0.001, Nagelkerke pseudo R**^**2**^ **=** **26.5%**			
Single (0 = no, 1 = yes)	1.39 (0.63)	4.01	0.028*
Housing at baseline (0 = stable, 1 = unstable)	1.80 (0.57)	6.07	0.001^*^
Constant	−2.88 (0.58)		
**Second model built by forward stepwise selection of treatment variables**^**T**^ **X**^**2**^ **(1)** **=** **7.132**, ***p*** **=** **0.008, Nagelkerke pseudo R**^**2**^ **=** **10.7%**			
Inpatient care (0 = no, 1 = yes)	1.47 (0.54)	4.34	0.007^*^
Constant	−1.84 (0.32)		
**Third model built by entering significant variables from models above X**^**2**^ **(3)** **=** **21.852, p** **<** **0.001, Nagelkerke pseudo R**^**2**^ **=** **30.7%**			
Single (0 = no, 1 = yes)	1.34 (0.65)	3.84	0.037^*^
Housing at baseline (0 = stable, 1 = unstable)	1.67 (0.58)	5.31	0.004^*^
Inpatient care (0 = no, 1 = yes)	1.09 (0.60)	2.98	0.068
Constant	−3.14 (0.63)		

*Prediction of housing at discharge (0 = stable, 1 = unstable) from complete data (N = 102) on all tested variables (see superscripts B and T in Table 2)*.

* *Significant at p < 0.05*.

## Discussion

This prospective observational study documents the importance of housing in the complex dynamics between social stressors and treatment response in pregnant and parenting women with problems related to methamphetamine use. Our real-world data have direct implications for clinical practice as we continue developing integrated care for this underserved population.

### Baseline Correlates of Housing

Psychiatric comorbidities and social complexities were frequent in our naturalistic sample of pregnant and parenting women, echoing the multiple burdens reported in association with methamphetamine use (19, 24). We previously found that comorbid attention-deficit hyperactivity disorder and depression jeopardized treatment engagement in integrated care (27). Yet in contrast to social challenges, psychiatric comorbidities were not significantly related to housing at discharge. As both treatment discontinuation and unstable housing predict substance use (30–32), our studies indicate that psychiatric comorbidities and social challenges are differentially associated with important determinants of treatment success.

Being single may be a key stressor as it predicted unstable housing at discharge even when adjusting for housing at admission. This aligns with a study in people injecting substances where a partnered relationship seemed conducive to attaining stable housing (28). Yet substance-using partners are influential in the use of methamphetamine (9, 13), and being single was significantly related to the other parent having a substance use disorder, which predicted unstable housing at discharge as did living apart from children. These data combined with the stresses of single parenting (10, 12–14) indicate that women could greatly benefit from interventions targeted at building relationships. Couple counseling should be offered and cover substance use, family planning, and parenting skills (19).

The majority of unstably housed women at discharge had a criminal record (prison sentence in over one-third), whereas almost two-thirds of stably housed patients had no record (prison sentence in one-sixth). Although there was no overall significant difference in criminal system involvement between these groups, incarceration history was previously associated with an increased likelihood of unstable housing and methamphetamine use (33). Thus, housing may be lost while imprisoned and prison sentences may hamper attaining stable housing. As employment rates were similarly low across stably and unstably housed women at discharge, prison sentences may thwart stable housing by factors other than lost income, such as lost community or additional stigma. Nonetheless, the low levels of employment highlight the general need for vocational education, training, and reintegration.

### Treatment Correlates of Housing

Twelve of 23 women who were unstably housed at admission (three of six homeless) achieved stable housing by discharge. Nine women transitioned from their own to supported transitional accommodation with children as they required intensive care that could not otherwise be provided after discharge from Mamadam.

The relations among housing at admission and discharge, being single, the other parent having a substance use disorder, mother-child separation, social work involvement, and inpatient care and rehabilitation show that women in difficult circumstances were more likely to access social and inpatient services, and to transition to inpatient rehabilitation. Stable housing would facilitate the use of outpatient social work to build community relationships, receive home support for childcare, and pursue employment. To deliver on this prospect, stronger partnerships with government and community agencies are needed to provide faster access to public housing and support for independent housing.

Housing at discharge was not significantly associated with adherence to or duration of Mamadam, or with prior withdrawal program participation. Yet less than one-third of unstably housed women at discharge had discontinued Mamadam and almost two-thirds had accessed a withdrawal program, whereas adherence and withdrawal program participation were below 50% in stably housed women at discharge. In mothers with substance use and/or other psychiatric disorders, accessing treatment was related to being single and having a low socioeconomic status (11). However, substance use treatment was inversely associated with attaining stable housing, even after adjustment for relationship status, employment status, and substance use (28). Completion of addiction treatment is associated with abstinence, less crime, and greater employment (32), whereas the relation to housing is mixed (30). These findings collectively suggest that current programs engage socially disadvantaged patients in care but are often not sufficient to improve housing conditions.

### Limitations

We used a dichotomous measure of housing stability as more women in supported transitional accommodation with children, living in others' homes, and without housing would have been required to gain insight into potential differences between these living arrangements. The lack of post-discharge data precludes claims regarding potential benefits of transitioning from own to supported transitional housing for some women. Moreover, our care concept and some of our findings (e.g., employment unrelated to housing at discharge) may not be readily transferable to other healthcare systems and communities in different social, financial, or legal contexts.

## Conclusion

Integrated services promise better care for pregnant and parenting women with problems related to methamphetamine use, yet challenges remain given the complex needs of this population. Single women and unstably housed women are particularly at risk of remaining in precarious living conditions, which threaten to drive them into heavier substance use and socioeconomic deprivation. Future work should consider developing and studying housing and relationship interventions, which may break this downward spiral.

## Data Availability Statement

The raw data supporting the conclusions of this article will be made available by the authors, without undue reservation.

## Ethics Statement

The studies involving human participants were reviewed and approved by the Ethics Committee at the Carl Gustav Carus Faculty of Medicine at the Technische Universität Dresden, Germany. Written informed consent for participation was not required for this study in accordance with the national legislation and the institutional requirements.

## Author Contributions

UZ, MP, and MS contributed to the development of the care concept. UZ, MP, MS, and BW were members of the care team. MP, UZ, LR, MK, and JP designed the study. UZ, MP, and JP obtained funding. LR, MK, MS, BW, MP, and UZ collected the data. JP analyzed and interpreted the data and wrote the manuscript. All authors contributed to the article and approved the submitted version.

## Funding

The German Research Foundation (Project Number 402170461, TRR 265) (34) supported this research and paid the article processing charge but was not involved in the conceptualization or conduct of the study, the collection, analysis, interpretation of data, the preparation of the manuscript, or the decision to submit it for publication.

## Conflict of Interest

The authors declare that the research was conducted in the absence of any commercial or financial relationships that could be construed as a potential conflict of interest.

## Publisher's Note

All claims expressed in this article are solely those of the authors and do not necessarily represent those of their affiliated organizations, or those of the publisher, the editors and the reviewers. Any product that may be evaluated in this article, or claim that may be made by its manufacturer, is not guaranteed or endorsed by the publisher.
